# Emergent *Vibrio parahaemolyticus* Gastroenteritis Outbreaks, New Zealand, 2019–2022

**DOI:** 10.3201/eid3208.260097

**Published:** 2026-08

**Authors:** Sarah Jefferies, Shevaun Paine, Jing Wang, Xiaoyun Ren, David Winter, Graham C. Fletcher, Anne-Marie Perchec-Merien, Jackie Wright

**Affiliations:** New Zealand Institute for Public Health and Forensic Science, Porirua, Wellington, New Zealand (S. Jefferies, S. Paine, J. Wang, X. Ren, D. Winter, J. Wright); New Zealand Institute for Bioeconomy Science Limited, Auckland, New Zealand (G.C. Fletcher); Ministry for Primary Industries, Wellington (A.-M. Perchec-Merien)

**Keywords:** *Vibrio parahaemolyticus*, bacteria, food safety, enteric infections, foodborne diseases, pandemics, New Zealand

## Abstract

We report the Trans-Pacific expansion of *Vibrio parahaemolyticus* pandemic clone sequence type 36 and emergence of sequence type 50 in Oceania, causing seafoodborne outbreaks in New Zealand. Unusual features of the outbreaks included diversity of the seafood sources and pathogenic strains and timing, occurring in both winter and summer seasons.

*Vibrio parahaemolyticus* is the most commonly reported cause of bacterial seafoodborne illness worldwide (*1*). Acute gastroenteritis (AGE) classically occurs within 36 hours of consuming raw or undercooked bivalve molluscan shellfish (e.g., mussels, oysters), which concentrate the thermal-sensitive bacterium through filter-feeding. In many places, including New Zealand, *V. parahaemolyticus* AGE is a legally notifiable disease prompting public health action for foodborne disease control (*2*).

*V. parahaemolyticus–*related illness has demonstrated major epidemiologic shifts over the past decade. Before the 2010s, AGE cases resulting from *V. parahaemolyticus* ingestion were mostly confined to endemic and outbreak-prone regions, such as Japan, China, and the northwest United States (*3*–*5*). However, global environmental changes (rising sea-surface temperatures), microbial genomic diversification, and emergence and increasing expansion of pandemic clone sequence type (ST) 3 and ST36 have led to emergence of *V. parahaemolyticus* illness in new areas (*3*,*5*,*6*). In 2012, researchers identified ST36, which was endemic to the Pacific Northwest United States, in human infections in northeast regions of the country (*5*). Outbreaks of *V. parahaemolyticus *ST36*–*related illness were subsequently identified in Spain and Peru (*3*,*5*). 

*V. parahaemolyticus* AGE has been a rare disease in Australasia and the Pacific Islands. New Zealand (population ≈5 million) has an extensive marine aquaculture industry and deep cultural connections with coastal marine ecology, including those related to indigenous Māori tikanga (cultural practices) (*7*). We describe emergence of *V. parahaemolyticus* AGE in Aotearoa New Zealand, including the distinctive epidemiologic features and microbial genomics relevant to outbreak cases.

## The Study

In response to a surge in clinical notifications in May 2019, we undertook 4 successive multidisciplinary *V. parahaemolyticus* AGE outbreak investigations (Table). This effort required enhanced epidemiologic investigation, establishment of national *V. parahaemolyticus* whole-genome sequencing capability (Appendix), and extensive environmental investigations, including food source traceback, testing of implicated seafoods, sea surface temperature measurements, and seafood supply chain inquiries. During January 2019–May 2022, 136 of the 182 cases entered into the national notifiable disease database had viable *V. parahaemolyticus* isolates referred to the New Zealand Institute for Public Health and Forensic Science: 90 outbreak-related and 46 background isolates (predominantly locally acquired [38/46] owing to COVID-19 border restrictions during the period) (*2*). We also identified 4 shellfish isolates from outbreak-implicated mussel farms, sampled by the New Zealand Institute for Bioeconomy Science and Ministry for Primary Industries in 2020 and 2021. We subjected all isolates to *tdh*/*trh*-toxin gene testing and whole-genome sequencing, comparing New Zealand strains to available international sequences (Appendix).

**Table:** Epidemiologic and molecular characteristics of *Vibrio parahaemolyticus* acute gastroenteritis cases linked to outbreaks in New Zealand, May 13, 2019–April 20, 2022*

Characteristic	All outbreaks	Outbreak 1	Outbreak 2	Outbreak 3	Outbreak 4
Notification date range; season(s)		2019 May 13–Jun 18; late autumn–winter	2020 Jun 08–Jul 27; winter	2021 Feb 09–Apr 28; late summer–autumn	2021 Nov 15–2022 Apr 20; late spring–autumn
No. cases (confirmed, probable)	117 (116 confirmed, 1 probable)	23 (23 confirmed)	16 (16 confirmed)	21 (21 confirmed)	57 (56 confirmed, 1 probable)
Sex, no. (%)					
M	60 (51)	14 (61)	10 (63)	10 (48)	26 (46)
F	57 (49)	9 (39)	6 (37)	11 (52)	31 (54)
Age, y, range (median)	17–90 (57)	23–80 (59)	38–77 (61)	17–83 (53)	17–90 (53)
Ethnicity, no. (%)					
Māori	53 (45)	15 (65)	8 (50)	10 (48)	20 (35)
Pacific peoples	11 (9.4)	NA	2 (13)	2 (9.5)	7 (12)
Asian	2 (1.7)	NA	NA	NA	2 (3.5)
European or Other	51 (44)	8 (35)	6 (38)	9 (43)	28 (49)
Cases by NZDep Index quintile,† no. (%)					
1 (least deprived)	17 (15)	2 (8.7)	1 (6.3)	4 (19)	10 (18)
2	12 (10)	0 (0)	1 (6.3)	3 (14)	8 (14)
3	24 (21)	4 (17)	1 (6.3)	4 (19)	15 (26)
4	25 (21)	7 (30)	4 (25)	4 (19)	10 (18)
5 (most deprived)	39 (33)	10 (43)	9 (56)	6 (29)	14 (25)
No. districts with cases; top 3 districts by case count (no.)	15; Canterbury (25), Bay of Plenty (15), Counties Manukau (13)	8; Bay of Plenty (7), Waitemata (6), Counties Manukau (5)	9; Counties Manukau (5), Waikato (3), Bay of Plenty (2)	7; Southern (7), Nelson Marlborough (5), Canterbury (4)	13; Canterbury (20), Northland (7), Bay of Plenty (6)
Hospitalized cases, no. (%)	43 (37)	8 (35)	7 (44)	5 (24)	23 (40)
ST, no./total (%)‡					
ST36	5/5 (100)	5/5 (100)	NA	NA	NA
ST50	NA	NA	7/7 (100)	4/4 (100)	16/23 (70)
Other ST	NA	NA	NA	NA	7/23 (30)
Cases with pathogen subtyping, no. (%)§	90 (77)	8 (35)	16 (100)	15 (71)	51 (89)
Cases with ST *tdh*/*trh* profile (no.)	ST50 *tdh*+/*trh*+ (70); ST50 *tdh*+/*trh*– (1); ST36 *tdh*+/*trh*+ (8); ST199 *tdh*+/*trh*+ (1); ST3 *tdh*+/*trh*– (1); ST8 *tdh*–/*trh*+ (1); ST55 *tdh*–/*trh*– (1); ST1140 *tdh*+/*trh*– (1); ST2549 *tdh*–/*trh*– (4); ST2903 *tdh*–/*trh*– (1) ¶; ST2904 *tdh*–/*trh*– (1) ¶	ST36 *tdh*+/*trh*+ (8)	ST50 *tdh*+/*trh*+ (16)	ST50 *tdh*+/*trh*+ (15)	ST50 *tdh*+/*trh*+ (39); ST50 *tdh*+/*trh*– (1); ST3 *tdh*+/*trh*– (1); ST8 *tdh*–/*trh*+ (1); ST55 *tdh*–/*trh*– (1); ST199 *tdh*+/*trh*+ (1); ST1140 *tdh*+/*trh*– (1); ST2549 *tdh*–/*trh*– (4); ST2903 *tdh*–/*trh*– (1)¶; ST2904 *tdh*–/*trh*– (1)¶
Implicated source(s) by case (no.)#	NA	Raw or part-cooked mussels (23)	Raw or part-cooked mussels (12), fish (4), shellfish unspecified (3), lobster (1)	Raw or part-cooked mussels (20), sea urchin (1)	Raw or part-cooked: oysters (17; 7 ST50), mussels (14; 10 ST50), sea urchin (8; 6 ST50), lobster (7; 7 ST50), pāua (5; 4 ST50), pipis (4; 4 ST50), clams (1; 1 ST50), shellfish unspecified (2; 0 ST50), fish (3; 3 ST50)
Supplier(s)	NA	Commercial to superstores (23)	Commercial to superstores (15), food premise (2), unknown (3)	Commercial to superstores (20), self-collected sea urchin (1)	Self-collected (38), store bought (17), unknown (6)
Coastal location(s)	NA	Coromandel region upper North Island mussel-growing area	Coromandel region upper North Island mussel-growing area	Marlborough region upper South Island mussel-growing area; North Island	New Zealand-wide North and South Island coastal regions

*NA, not applicable; NZDep, New Zealand Deprivation Index; ST, sequence type. †NZDep 2018 quintile whereby 1 is least and 5 is most socioeconomically deprived. ‡No. hospitalized cases with specified core-genome multilocus sequence type infection out of the total no. hospitalized cases with available ST results. §*V. parahaemolyticus* subtyping results (*tdh*/*trh* toxin gene profile and ST) for all cases with an isolate referred for subtyping. ¶Novel strains that were assigned sequence types during the investigation. #No. cases reporting consuming specified types of seafood within the incubation period for illness; some cases reported consuming more than 1 type of seafood so counts may exceed number of cases. For multi-ST outbreak 4, no. cases reporting specific seafood exposures is also presented for ST50 cases in parentheses.

**Figure 1:**
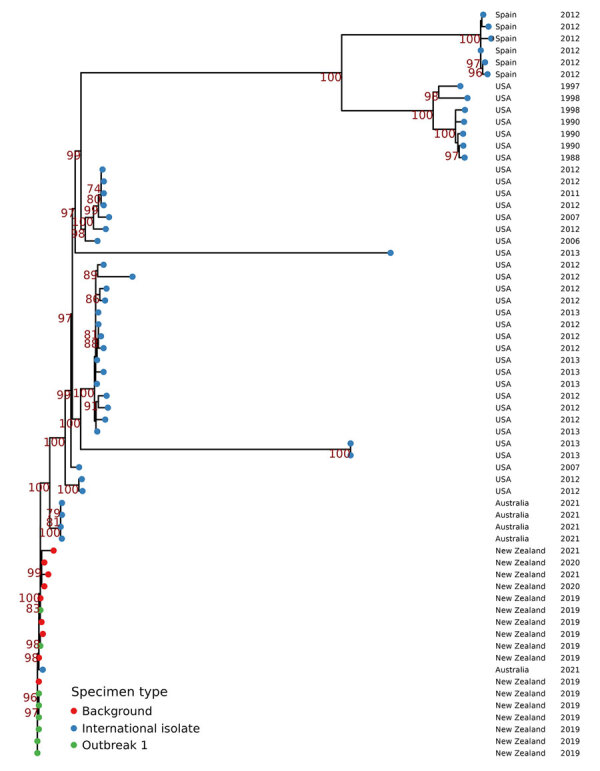
Maximum-likelihood phylogenetic tree of sequence type 36 isolates for study of emergent *Vibrio parahaemolyticus* gastroenteritis outbreaks, New Zealand, 2019–2022. Tree was constructed using core-genome single-nucleotide polymorphism alignment of all publicly available New Zealand and international *V. parahaemolyticus* sequence type 36 isolates detected during 1990–2021. Bootstrap values are calculated from 2000 trees; only bootstrap values >70% are shown in red.

Investigations revealed the first *V. parahaemolyticus* AGE outbreak was caused by pandemic clone ST36, widely distributed to cases through commercially supplied New Zealand green-lipped mussels (Table). This finding represented Trans-Pacific expansion of *tdh+/trh+* ST36 to Oceania; New Zealand isolates clustering (<30–50 single-nucleotide polymorphisms [SNPs]) with a Pacific Northwest/northeast USA lineage detected since 2007 (Figure 1). An ST36 infection occurred in April 2019 in a background case and seafood area distinct from the winter outbreak source. The close clustering (<5 SNPs) of this isolate with outbreak isolates and those causing sporadic illness thereafter demonstrated ST36’s high clonality and wide dispersal in New Zealand. Five closely related clinical isolates were also later reported in Australia in 2021.

**Figure 2:**
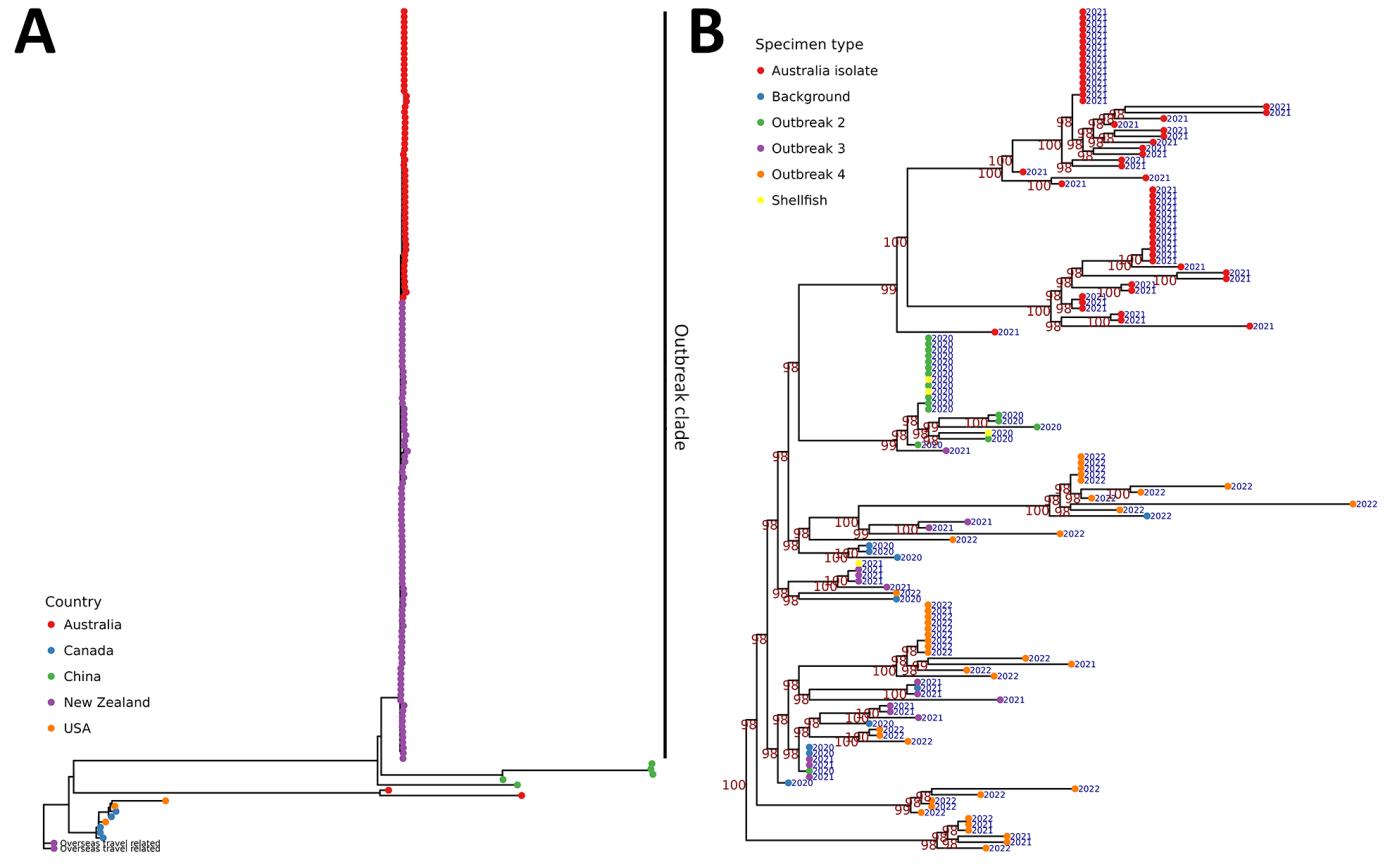
Maximum-likelihood phylogenetic tree of sequence type 50 isolates for study of emergent *Vibrio parahaemolyticus* gastroenteritis outbreaks, New Zealand, 2019–2022. Tree was constructed using core-genome single-nucleotide polymorphism alignment of all publicly available New Zealand and international *V. parahaemolyticus* sequence type 50 isolates detected during 1997–April 2022. The New Zealand sequence type 50 outbreak clade, which includes Australia isolates, is indicated. B) Magnification of the New Zealand *V. parahaemolyticus* sequence type 50 outbreak clade maximum-likelihood phylogenetic tree, by specimen type and sample collection year. Bootstrap values are calculated from 2,000 trees; only bootstrap values >70% are shown in red.

Two subsequent outbreaks in winter 2020 and summer 2021, also associated with commercially supplied mussels, represented the emergence of pathogenic *tdh+/trh+* ST50 in New Zealand (Table; Figure 2). We noted clinical and mussel-derived outbreak isolates to be closely related to background ST50 infections associated with geographically dispersed seafood growing areas, suggesting a bloom of clonal ST50 in New Zealand’s marine environment in 2020 replaced pandemic ST36 predominance. Internationally, ST50 is relatively uncommon, and New Zealand ST50 was genetically distinct from most international isolates, except those later emerging in an Australia oysterborne outbreak (*8*). Australia’s 2021 samples formed a monophyletic group, with some closely related to New Zealand samples (Figure 2, panel B). Those results are insufficient to demonstrate dispersal from New Zealand because separate incursions from a common source may have occurred. Nevertheless, the genomic epidemiology illustrates the interconnected nature of international marine ecosystems and emerging risks. Suspected mechanisms for the extensive global dispersal of related clones have included the international shellfish trade and contaminated ballast from cargo ships (*5*). Environmental conditions associated with aquaculture also may promote the evolution of *Vibrio *spp. virulence (*1*). Our findings suggest the need for further research into New Zealand ST50’s emergence.

The occurrence of wintertime outbreaks is unusual when compared with similarly temperate areas overseas (*1*). Sea surface temperatures >15°C, salinity, and rainfall can affect *V. parahaemolyticus* abundance and pathogenicity in seafood (*1*,*9*,*10*). Rising seawater temperatures in New Zealand may be contributing to recent trends in seafoodborne illness (*11*); sea surface temperatures recorded at mussel farms during winter outbreaks averaged 16.3°C in 2019 and 18°C in 2020. Some studies have also suggested higher relative prevalence of pathogenic *tdh+/trh+ V. parahaemolyticus* during colder temperatures (*9*,*12*). We identified a third *tdh+/trh+ V. parahaemolyticus* type during our investigations*, *ST199, isolated from cases who had consumed mussels associated with sea surface temperatures <16°C, suggesting this strain may have pathogenic preponderance at lower temperatures. Another hypothesized contributor to the occurrence of winter outbreaks is consumption of raw New Zealand green-lipped mussels as a naturopathic arthritis remedy; however, investigators did not collect information from cases related to reasons for mussel consumption (*13*).

Outbreaks 3 and 4 occurred during more typical summer months. Outbreak 4 showed diversity with regard to both implicated seafood sources and *V. parahaemolyticus* strains, and cases became predominantly associated with self-collection of wild shellfish (Table). ST50 from those outbreaks showed multiple distinct subclusters embedded within the wider diversity of historical clinical and environmental isolates (Figure 2, panel B). Outbreak 4 was thus likely driven by a surge of ST50 lineages already established across New Zealand, possibly triggered by an environmental amplification event. The summer of 2021–22 was New Zealand’s fifth warmest recorded summer; daily sea surface temperatures reached 4°C–5°C above average (*14*).

The apparent mix of pathogen-specific virulence and environmental factors in contributing to the emergence of epidemic *V. parahaemolyticus* disease is in keeping with experiences reported in Peru associated with El Niño (warmer, wetter) climatic events and arrivals of pandemic *Vibrio *spp. (*3*). In that context, the expansion of monitoring of harvest sites could support the early detection of pathogenic *V. parahaemolyticus* events. In response to New Zealand’s outbreaks, the Ministry for Primary Industries amended national regulations for commercial shellfish growing areas (*15*).

New Zealand’s experience with *V. parahaemolyticus*–associated outbreaks also has implications for health equity. Outbreaks disproportionately affected persons of Māori ethnicity (45% of cases and 47% of hospitalizations, but 17% of New Zealand’s population), as well as people of lower socioeconomic status (Table). Supporting Māori and local communities in the safe collection of seafood as a cultural activity and inexpensive food source is critical, including exploring novel methods of environmental monitoring and messaging that highlights the diversity of potentially affected seafoods.

## Conclusions

We report Trans-Pacific expansion of pandemic ST36 *V. parahaemolyticus* to Oceania and emergence of pathogenic ST50, during both cold and warm seasons, associated with distributed musselborne outbreaks, a multisource outbreak of ST50, and uncommon STs affecting varied seafoods. Environmental drivers of *Vibrio* disease emergence, particularly increasing global climatic changes, require enhanced surveillance, policy, and research responses integrating One Health approaches and tailored local community considerations. Further integrated research should explore virulence, risk factors, and novel methods of environmental monitoring. Our investigations led to greater standardization of local diagnostic practices (initially limited by diverse methodologies, culture-free testing, and incomplete referrals for subtyping), investment in prospective genomic *V. parahaemolyticus* surveillance, environmental surveillance enhancements, and changes to food safety regulations. 

Appendix Additional information for emergent *Vibrio parahaemolyticus* gastroenteritis outbreaks, New Zealand, 2019–2022
