# Supplementary material for: Emergent Vibrio parahaemolyticus Gastroenteritis Outbreaks, New Zealand, 2019–2022
Source: Emerg Infect Dis. 2026 Aug;32(8):1345–9. doi: 10.3201/eid3208.260097 (PMC13426881; doi:10.3201/eid3208.260097)
Supplement: Appendix — Additional information for emergent Vibrio parahaemolyticus gastroenteritis outbreaks, New Zealand, 2019–2022 [file 26-0097-Techapp-s1.pdf]

Article DOI: http://doi.org/10.3201/eid3208.260097

EID cannot ensure accessibility for supplementary materials supplied by authors. Readers who have difficulty accessing supplementary content should contact the authors for assistance.

Emergent *Vibrio parahaemolyticus* Gastroenteritis Outbreaks, New Zealand, 2019–2022

Appendix

Additional Methods

Outbreaks were defined by periods of increased disease prevalence relative to baseline trends, with outbreak cases classified as either: confirmed – case isolate is the predominant outbreak sequence type (ST), or the ST is non-predominant or is unavailable but the case has a common source exposure (i.e., specific New Zealand-origin seafood) as a predominant ST laboratory-confirmed case; or probable – case consumed raw/undercooked seafood of suspect common outbreak source but epidemiologic information is limited and the infection ST is unknown or non-predominant. Cases were excluded by a history of overseas travel within the incubation period or if the implicated seafood was overseas-sourced.

Viable *Vibrio parahaemolyticus* isolates underwent *tdh*/*trh*-toxin gene testing (*1*) and whole genome sequencing (WGS). For WGS performed by the New Zealand Institute for Public Health and Forensic Science, genomic DNA were extracted using the chemagic 360 extraction platform (PerkinElmer, Waltham, MA, USA) or DNeasy Blood and Tissue method (Qiagen, Hilden, Germany). DNA libraries were created using the Nextera XT DNA sample preparation kit (Illumina, San Diego, CA) and paired-end sequencing of 2×150 bp was performed on the NextSeq 550 platform (Illumina, San Diego, CA). Sequencing quality assessment and initial analysis were undertaken using an in-house pipeline using fastp (v0.20) (*2*) for sequence statistics, SKESA (v2.3.0) (*3*) for assembly mlst (v2.16.2) (*4*) to assign 7-gene Multi Locus Sequence Typing (7-gene MLST) and Centrifuge (v1.0.4b) for species identification (*5*). Reads passing QC were characterized using nullarbor2: 'Reads to report' for public health and clinical microbiology pipeline (v2.0.20181010) including

abricate (v0.8.13) for detection of virulence factor. Sequences with novel alleles were uploaded to pubMLST for ST designation (*6*).

The genetic relationships between all isolates were investigated using a core genome MLST (cgMLST) method, based on the 2254 genetic loci in the publicly available *V. parahaemolyticus* cgMLST scheme using Chewbacca (v3.2.0) (*6*,*7*).

Initial single nucleotide polymorphism (SNP)-based analysis of the 2020 outbreak identified that a closely related reference genome was essential for epidemiologically informative SNP-level analyses. A ST50 reference genome was generated for a local isolate (SAMN44062662) using long read sequencing (MinION, Oxford Nanopore Technologies, Oxford, England). Separate SNP analyses were carried out for the ST50 genomes (using the novel reference genome) and ST36 genomes (using National Centre for Biotechnology Information (NCBI) refseq sequences for each chromosome:NZ_CP026041.1 and NZ_CP026042.1). For each dataset, core-SNPs were identified using Snippy4 (v4.3.6) (*8*). IQ_TREE (v2.0.6) (*9*) was used to estimate a phylogenetic tree from the resulting core-SNP alignment and pairwise SNP distances were calculated using snp-dists (v 0.6.3) (*10*). The SNP distances matrix was used to define genomic clusters using the single-linkage clustering algorithm with 5-SNP threshold.

New Zealand data (NCBI Bioproject PRJNA1063762) were analyzed with international sequences. A search of publicly available data in July 2019 identified 41 ST36 sequences from the USA and Spain (*11*,*12*). A repeat search in March 2025 identified five from Australia collected in 2021. Seventy international sequences were identified for ST50, including 57 sequences from an Australian outbreak in late 2021 (*13*).

References

1. Tada J, Ohashi T, Nishimura N, Shirasaki Y, Ozaki H, Fukushima S, et al. Detection of the thermostable direct hemolysin gene (*tdh*) and the thermostable direct hemolysin-related hemolysin gene (*trh*) of *Vibrio parahaemolyticus* by polymerase chain reaction. Mol Cell Probes. 1992;6:477–87. PubMed https://doi.org/10.1016/0890-8508(92)90044-X

2. Chen S, Zhou Y, Chen Y, Gu J. fastp: an ultra-fast all-in-one FASTQ preprocessor. Bioinformatics. 2018;34:i884–90. PubMed https://doi.org/10.1093/bioinformatics/bty560

3. Souvorov A, Agarwala R, Lipman DJ. SKESA: strategic k-mer extension for scrupulous assemblies. Genome Biol. 2018;19:153. PubMed https://doi.org/10.1186/s13059-018-1540-z

4. Seeman T. MLST. Melbourne: University of Melbourne; 2020 [cited 2020 Jul 30]. https://github.com/tseemann/mlst

5. Kim D, Song L, Breitwieser FP, Salzberg SL. Centrifuge: rapid and sensitive classification of metagenomic sequences. Genome Res. 2016;26:1721–9. PubMed https://doi.org/10.1101/gr.210641.116

6. Jolley KA, Bray JE, Maiden MCJ. Open-access bacterial population genomics: BIGSdb software, the PubMLST.org website and their applications. Wellcome Open Res. 2018;3:124. PubMed https://doi.org/10.12688/wellcomeopenres.14826.1

7. Silva M, Machado MP, Silva DN, Rossi M, Moran-Gilad J, Santos S, et al. chewBBACA: A complete suite for gene-by-gene schema creation and strain identification. Microb Genom. 2018;4:e000166. PubMed https://doi.org/10.1099/mgen.0.000166

8. Seeman T. Snippy: fast bacterial variant calling from NGS reads. Melbourne: University of Melbourne; 2019 [cited July 2019]. https://github.com/tseemann/snippy

9. Nguyen LT, Schmidt HA, von Haeseler A, Minh BQ. IQ-TREE: a fast and effective stochastic algorithm for estimating maximum-likelihood phylogenies. Mol Biol Evol. 2015;32:268–74. PubMed https://doi.org/10.1093/molbev/msu300

10. Seeman T. SNP-dists: Pairwise SNP distance matrix from a FASTA sequence alignment. Melbourne: University of Melbourne; 2019 [cited June 2022]. https://github.com/tseemann/snp-dists

11. Martinez-Urtaza J, Trinanes J, Abanto M, Lozano-Leon A, Llovo-Taboada J, Garcia-Campello M, et al. Epidemic Dynamics of *Vibrio parahaemolyticus* Illness in a Hotspot of Disease Emergence, Galicia, Spain. Emerg Infect Dis. 2018;24:852–9. PubMed https://doi.org/10.3201/eid2405.171700

12. Martinez-Urtaza J, van Aerle R, Abanto M, Haendiges J, Myers RA, Trinanes J, et al. Genomic Variation and Evolution of *Vibrio parahaemolyticus* ST36 over the Course of a Transcontinental Epidemic Expansion. MBio. 2017;8:e01425-17. PubMed https://doi.org/10.1128/mBio.01425-17

13. Fearnley E, Leong LEX, Centofanti A, Dowsett P, Combs BG, Draper ADK, et al. *Vibrio parahaemolyticus* Foodborne Illness Associated with Oysters, Australia, 2021-2022. Emerg Infect Dis. 2024;30:2271–8. PubMed https://doi.org/10.3201/eid3011.240172

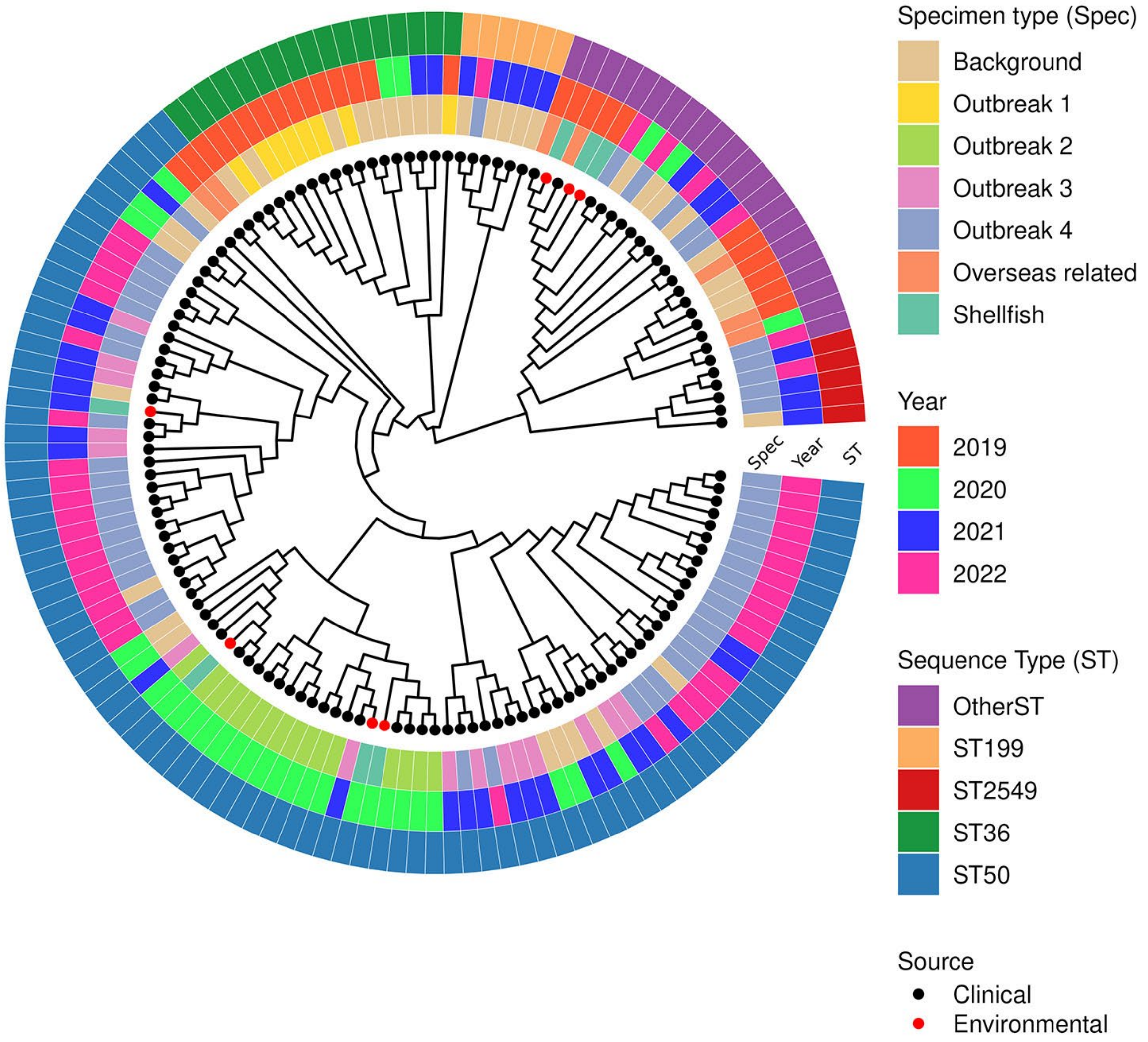

Appendix Figure. Neighbor-joining distance tree based on core genome multilocus sequence typing of all sequenced *Vibrio parahaemolyticus* causing acute gastroenteritis in New Zealand, January 2019–May 2022. Branch lengths are not proportional to genetic distance.
